# Nano Delivers Big: Designing Molecular Missiles for Cancer Therapeutics

**DOI:** 10.3390/pharmaceutics3010034

**Published:** 2011-01-13

**Authors:** Sachin Patel, Ashwin A. Bhirde, James F. Rusling, Xiaoyuan Chen, J. Silvio Gutkind, Vyomesh Patel

**Affiliations:** 1 Oral and Pharyngeal Cancer Branch, National Institute of Dental and Craniofacial Research, National Institutes of Health, Bethesda, MD 20892, USA; 2 Laboratory of Molecular Imaging and Nanomedicine, National Institute of Biomedical Imaging and Bioengineering, National Institutes of Health, Bethesda, MD 20892, USA; 3 Department of Cell Biology, University of Connecticut Health Center, Farmington, CT 06032, USA; 4 Institute of Materials Science, University of Connecticut, Storrs, CT 06269, USA; 5 Department of Chemistry, University of Connecticut, Storrs, CT 06269, USA

**Keywords:** nano-material, cancer, drug delivery, nano-toxicity, carbon nanotube, siRNA, gene delivery

## Abstract

Current first-line treatments for most cancers feature a short-list of highly potent and often target-blind interventions, including chemotherapy, radiation, and surgical excision. These treatments wreak considerable havoc upon non-cancerous tissue and organs, resulting in deleterious and sometimes fatal side effects for the patient. In response, this past decade has witnessed the robust emergence of nanoparticles and, more relevantly, nanoparticle drug delivery systems (DDS), widely touted as the panacea of cancer therapeutics. While not a cure, nanoparticle DDS can successfully negotiate the clinical payoff between drug dosage and side effects by encompassing target-specific drug delivery strategies. The expanding library of nanoparticles includes lipoproteins, liposomes, dendrimers, polymers, metal and metal oxide nano-spheres and -rods, and carbon nanotubes, so do the modes of delivery. Importantly, however, the pharmaco-dynamics and –kinetics of these nano-complexes remain an urgent issue and a serious bottleneck in the transition from bench to bedside. This review addresses the rise of nanoparticle DDS platforms for cancer and explores concepts of gene/drug delivery and cytotoxicity in pre-clinical and clinical contexts.

## 1. Introduction

Nanotechnology is, in part, a science of synthesizing molecular sized materials that can range from a few nanometers to micrometers that are invisible to the human eye [1-3]. At this nanoscale, the tensile strength, opto-electrical properties, and surface chemistry of materials become radically changed. These features, if successfully exploited, could revolutionize medicine [4,5]. The application of nanotechnology in medicine is now known as nanomedicine, and the drug formulations using the nanomaterials are referred to as nanoformulations [6-8].

Despite recent progress in decreasing mortality, cancer still ranks as the second most frequent cause of death in the United States [9]. In this regard, advances in nanoformulated drug delivery systems (DDS) hold great promise for improved cancer patient outcomes. These DDS have been designed with formulations that were initially based upon drugs loaded into liposomes or polymer nanoparticles to improve their pharmacological properties and therapeutic outcome for parenterally administered drugs [10]. Using this approach, several anti-cancer DDS have now been approved for clinical use and have already greatly impacted oncological therapeutics [11]. More recently, research has moved toward development of related systems based on metal, metal oxide, and carbon nanoparticles which promise to solve some of the stability and toxicity problems of liposomal and polymer DDS [12-14]. These new drug delivery systems for cancer treatment with potentially reduced toxic side effects have enabled the development of improved therapeutic regimens with existing drugs that are currently standard-of-care. Examples of these delivery systems feature passive and active targeting, which essentially takes advantage of characteristic tumor features that allow nano-sized drug delivery systems to accumulate in the target area of the cancer; allow for superior cancer therapeutics by virtue of efficient cell entry; and lower toxicity. In this review, we describe examples of key developments in the area of cancer drug delivery systems using nanomaterials.

## 2. The Need for Nano

The arsenal of chemotherapeutic drugs used against most cancers is sufficiently potent to diminish their growth – that is, provided the drugs reach their desired location with high fidelity and efficiency. Owing to the non-specific and cytotoxic nature of first-line anti-cancer drugs, physicians are often forced to balance dosage with painful patient side effects. This war of attrition—a question of which cells are last to die, normal or cancerous—is further complicated by the high mutation frequency of certain cancers. As a result of a weakened drug regimen, multi-drug resistant cancer cell populations are more likely to evolve [15-17].

These shortcomings, among others, have led to a call for targeted drug delivery strategies that deliver a greater payload of drugs to the tumor site and limit the damage to normal, non-cancerous tissue [18]. The rapidly advancing field of nanomedicine has produced nanoparticle platforms that can ultimately function as molecular missiles, capable of delivering not only drugs but also genetic material, and acting as cancer-killing and imaging-contrast agents. The once small class of drug delivery vehicles that included only liposomes and polymers now comprises dendrimers, metallic and metal oxide nano-beads/shells, carbon nanotubes, and lipoproteins (Table 1) [19,20]. In a similar way, seek-and-destroy methods utilized are as variegated as their apparatuses and include receptor-mediated endocytosis, magnetically-directed localization, pH and thermal sensitive drug release, and external laser ablation, among others [21].

Though their versatility is unarguable, nanoparticle DDS toxicity still remains an impasse that researchers are attempting to navigate. Single-wall carbon nanotubes are hydrophobic in their natural pristine state, and unless oxidized they generally remain uncharged and neutral and susceptible to aggregating that can lead to arterial blockage in the lungs and kidneys of mice [37]. To address questions of biodistribution and toxicity, recent studies have demonstrated that when carbon nanotubes are oxidized to shorten them and to add negatively charged groups, and if these are then coated with solubilizing polymers [38] or various proteins [39], the nanotubes are much less toxic [40]. These treatments effectively eliminate aggregation, increase blood circulation time and eliminate induced interferon responses. Notwithstanding problems that still need to be solved, nanoparticles are versatile, multifunctional platforms with far-reaching and quickly materializing biological potential [41-43].

## 3. Nucleic Acid Delivery

The lipid bilayer of the cell membrane acts as a biological wall against foreign, pathogenic nucleic acids, and prevents therapeutic delivery of small interfering RNA (siRNA) or plasmid DNA [44]. Several viral and polymeric nanocapsules, cationic liposomes and, non-viral vectors (lipoplexes, polyplexes and inorganic nanoparticles) have been developed that can actively cross the lipid membrane and deliver nucleic acid cargos with relative ease and efficiency and with limited toxicity *in vitro*. While the library and efficiency of *in vitro* transfection and infection methods expands and improves, there is mounting clinical uncertainty with regards to gene transfer and therapy methods in living systems. Such *in vivo* therapies must not only selectively infiltrate the target cells but also do so without inducing inflammatory responses [45-47]. Several gene therapy alternatives exist: most seek to incorporate synthetic DNA into the host genome, a process that requires additional penetration of the nuclear envelope; others engage RNA-interference, the mechanism of action by which siRNA sequences binds to target messenger RNA and initiates its degradation. Historically, viral vectors were the preferred delivery mode, but preliminary clinical studies triggered fatal inflammatory reactions in one patient and leukemogenesis in another, diverting efforts into synthetic, less immunogenic vector systems able to be systemically administered [48]. Examples include cyclodextrins with transferrin used as a targeting ligand [49]; atelocollagen, a highly purified type I collagen that is free of immunogenic telopeptides and, when complexed with siRNA to form nanoparticles, these have been used to deliver with efficiency and little immunogenicity to bone metastatic tumors *in vivo* [50]; logic-embedded vectors [51] and microparticle carriers consisting of mesoporous silicon, allowing for a sustained release of second-stage neutral nanoliposomes (1,2-dioleoyl-*sn*-glycero-3-phosphatidylcholine (DOPC)), and used for delivering siRNA to the target, a cancer related receptor (EphA2 encoding ephrin type A receptor 2) and, resulting in a robust antitumor effect, without toxicity, in orthotopic models of ovarian cancer [52].

Like their viral predecessors, synthetic nano-vectors must fulfill two obligations: They must (1) efficiently and systematically traverse the cellular membrane, and (2) remain in circulation for a long enough time to do so. Moreover, the need to minimize nanoparticle toxicity must be balanced with the equally important need for vectors to maximize their interactions with cell-surface receptors. Following intracellular localization, the nucleic acid cargo must be efficiently cleaved and safely transported to avoid endosomal or nuclease degradation. If the target is the nucleus, as in the case of genetic introduction, the foreign DNA must traverse another barrier, the nuclear membrane [53]. Conversely, a siRNA mechanism of action, though difficult to deliver, represents a more facile and clinically effective approach to gene therapy and transfer. Because siRNA acts upon mRNA transcripts released into the cytoplasm, the additional rate-limiting step of nuclear localization is avoided [6,7].

**Figure 1.**
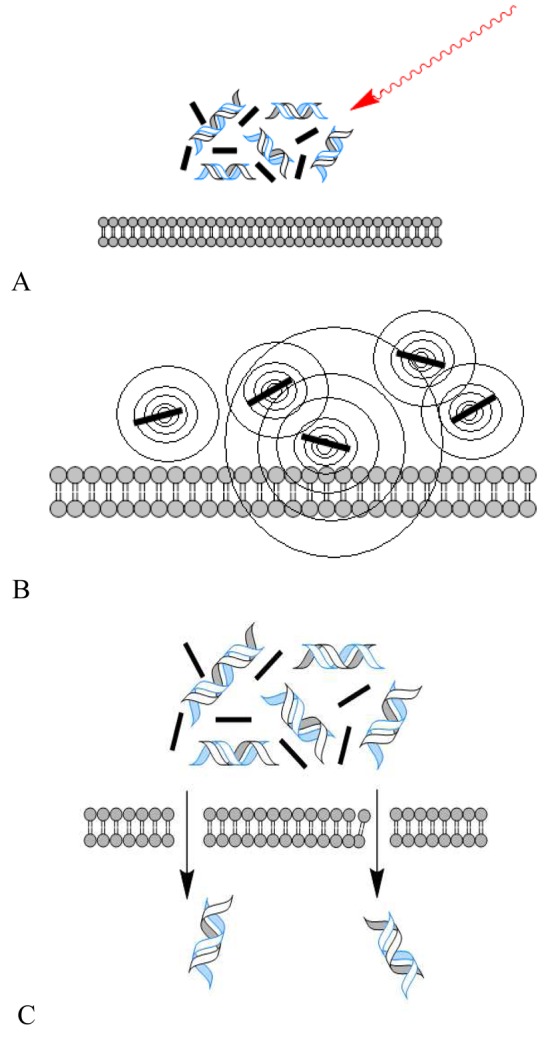
Laser activation of carbon black creates membrane perforation. (**A**) Femtosecond laser pulses applied to cells incubated with foreign DNA/carbon black. (**B**) Laser activation generates small acoustic shock-waves which disrupt the cellular membrane and create holes. (**C**) DNA is now able to traverse the cell membrane.

To bypass some of the limitations of current delivery methods, most notably host-immune rejection, and a need for greater efficiency of intracellular delivery of therapeutic agents, a recent study by Chakravarty *et al.* demonstrated a novel approach of membrane traversal, using carbon black nanoparticles (25–200 nm aggregates) activated by femtosecond laser pulses [53]. Drawing upon previously published data that such laser excitation results in carbon-steam reaction leading to consumption of carbon black and, generating local, highly controlled acoustic shock waves that create nanoscale holes in the lipid bilayer, the authors were able to deliver small molecules, proteins such as bovine serum albumin, and DNA into different model cell types. Contrary to the more physical, direct methods of electroporation, ultrasound, and laser irradiation, laser-activated carbon black nanoparticles are able to deliver small and potentially therapeutic molecules efficiently without greatly compromising cell viability (Figure 1 A-C).

A major roadblock to gene delivery occurs in the transition from *in vitro* conditions to *in vivo* models. Because siRNA-delivery relies principally upon the identification of a target cell population, it is nearly impossible to recapitulate systemic administration of the siRNA-nanoparticle vector *in vitro* as well as quantitatively evaluate clinical efficacy. Transgenic mice are one solution to the dilemma, having already been used to assess whole-body siRNA pharmacodynamics [54,55]. The luciferase transgenic mouse model is already established, and has allowed characterization of siRNA delivery dynamics via high-throughput, bioluminescent imaging. In this context, Tao *et al.* elegantly devised a novel process that enables evaluation of target-specific delivery of siRNA through the generation of a mouse model with liver specific expression of firefly luciferase [56]. They employed a group of lipid nanoparticles (LNPs) with different consistencies of cationic lipid, polyethylene glycol (PEG) lipid, and cholesterol as delivery vehicles, with luciferase siRNA (lucR) as the “drug payload.” Following systemic administration of 3 mg/kg of siRNA, *ex vivo* analyses (collection of mRNA and analysis using TaqMan qRT-PCR assay) was collated with the noninvasive, bioluminescence data. Both forms of analyses showed a dose-dependent repression of luciferase at 24, 72, and 96 hours after tail vein injection [56].

Additionally, recently published data by Davis *et al.* revealed the efficacy of drug delivery platforms in ferrying siRNA through the blood-waterways of the body [57]. As the first siRNA clinical trial to use a targeted nanoparticle-delivery system, the study confirmed the uptake of the siRNA and subsequent downregulation of the target protein (RRM2, a protein that forms the second subunit of the ribosome). The molecular schematic consisted of four elements: (1) a linear, cyclodextrin-based polymer (CDP), (2) the ligand of human transferrin protein (hTf) decorating the polymer's surface, (3) and (4) siRNA specific to the *RRM2*. Each patient (a total of three), who all presented with late stage metastatic melanoma and were refractory to standard-of-care therapies, received 18, 25, and 30 mg/m^2^, respectively, of siRNA. Notably, *ex vivo* analysis of the biopsies revealed a dose-dependent reduction of *RRM2* mRNA and protein product (confirmed through qRT-PCR and Western blot analysis) in the cancerous tissue [57,58]. Altogether, the increased efficacy and translational relevance of targeted gene therapy is becoming more and more apparent. Facilitated by nanoparticles—ranging from lipid structures to polymer-based compounds to carbon nanotubes—siRNA delivery is becoming an increasingly viable gene-specific, tissue-specific therapeutic choice.

Although synthetic DNA delivery systems are safe and versatile, they nonetheless remain inefficient. To address this, several studies have reported the potential use of nanoparticles with plasmid DNA delivery. Singh *et al.* developed nanotube-based gene delivery vectors, where several forms of functionalized nanotubes were able to condense plasmid DNA on to their surfaces. The study found that the charge density and the large surface area were critical parameters for this interaction, and these complexes were successfully used to deliver to the target cells, and the DNA facilitated gene expression [59]. Additionally, biodegradable polymers that include water-soluble cationic polymers and micro- and nanoparticles, have also been developed as efficient carriers of plasmid DNA, and once inside the cells, the carriers undergo degradation and the plasmid DNA is released into the cytosol [29].

## 4. Drug Delivery and Target Specificity

Cancer stands out among human diseases for its unique transformative abilities, rendering protein expression, and to some extent, entire cell behavior radically different from the normal counterpart. Whereas normal, non-cancerous cells may display a small numbers of growth factor receptors, cancer cells usually are decorated with large surface concentrations of receptors designed to maximize ligand-binding and intracellular signal initiation, providing an increased growth advantage [60]. For this reason, nanoparticles conjugated with the ligand partners to their cognate receptors are drawn towards the cancer cells with greatly enhanced affinity.

**Figure 2.**
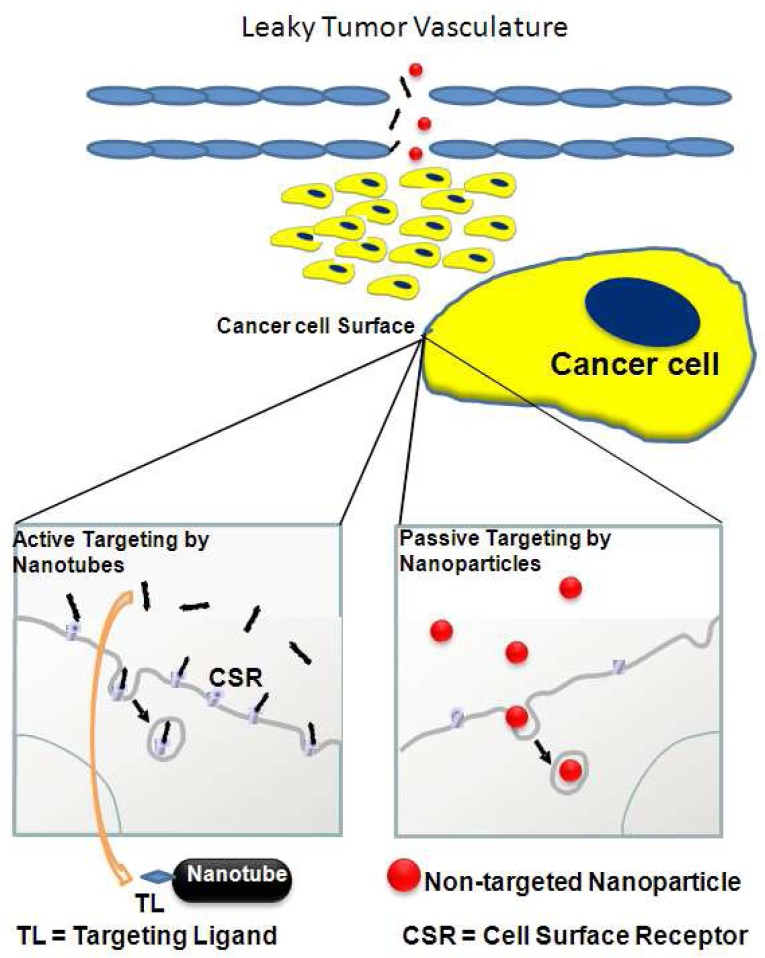
Active *versus* passive targeting in nanoparticle localization. **Active Targeting** (*left*): Ligand (antibody/peptide) driven localization, relying upon cancer surface receptor (CSR) mediated endocytosis. **Passive Targeting** (*right*): Enhanced permeability and retention (EPR) driven cellular localization, relying upon fluid endocytosis.

Two main modes currently prevail for nanoparticle administration to the cancer site, one of which does not require surface identification (Figure 2, right box). This mode of delivery, termed passive targeting, relies on abnormal large gap junctions in the endothelium of tumor blood vessels. This so-called enhanced permeability and retention (EPR) effect depends upon particle size, shape, and composition. Through this feature, nanoparticles gradually accumulate at the target site. Passive targeting, though, is limited in its usefulness for several reasons [18,21]. First, the EPR effect is highly dependent upon the location of the tumor and is strengthened or weakened by the presence or absence of adjacent blood vasculature networks. EPR's greatest deficiency is exposed when looking at the vasculature signature of solid tumors. In such cases, tumor vessels are poorly perfused with blood and are rendered dysfunctional, leading to poor penetration. Coupled with the high interstitial pressure of the vessels, which limits nanoparticle extravasation, passive diffusion is nearly impossible [21]. However, active targeting (Figure 2, left box), most often with a homing ligand that recognizes a surface receptor protein, compensates for these limiting factors. Among the tumor targeting moieties, include ligands (essentially defined as small molecule that binds specifically to a larger molecule), such as antibodies, and peptides that have strong affinity to their cognate, cellular binding partners, for example, tumor antigens, cell surface receptors, and tumor vasculature [18].

**Figure 3.**
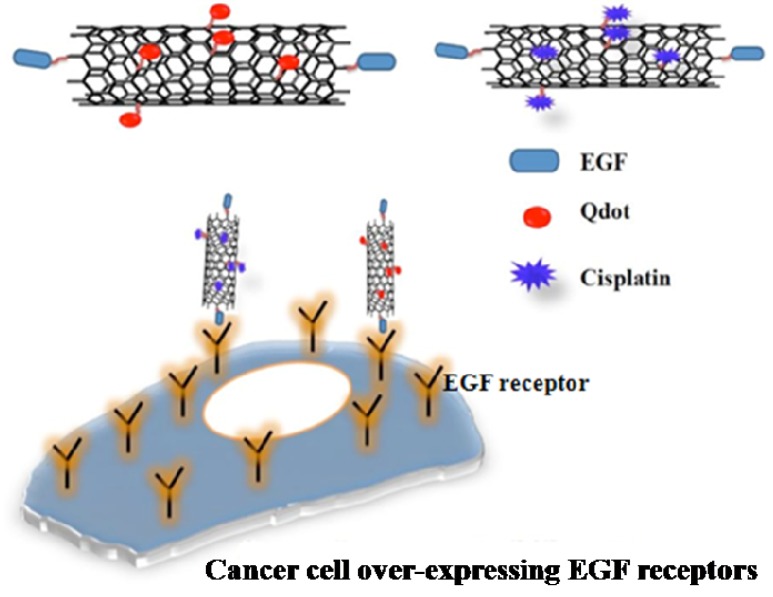
EGF directed killing of cancer cells using single walled carbon nanotube (SWCNT)-cisplatin delivery vector. Nanotubes coated with EGF ligand bind to the cognate EGF receptor on the cancer cell surface and internalize via receptor-mediated endocytosis. Quantum dot nanoparticles (Qdots) allow detection of the nanotubes.

Our research team recently demonstrated [61] the efficacy of EGF-cisplatin-conjugated single walled carbon nanotube (SWCNT) complexes in reducing tumor growth in mice (Figure 3). Briefly, EGF and cisplatin molecules were covalently linked to the surface of oxidized, shortened single-walled carbon nanotubes, creating molecular missiles that targeted an EGFR-overexpressing human head and neck cancer cell line. Strikingly, subsequent *in vivo* systemic administration of the bioconjugates (via tail vein injection) was found to retard growth of flank xenografts on athymic nude mice. Complexes lacking the EGF ligand showed little to no effect in the same set of *in vivo* experiments [61,62]. Luminescent quantum dot nanoparticles (Qdots) were used to track the fate of these nanotubes with optical imaging.

Targeted therapies involving nanoparticle vehicles, most notably liposomes have been in clinical use for some time [63]. Importantly, these liposomes with surfaces particularly amendable to surface conjugation and biological targeting have not only enhanced specificity, but also reduced toxicity, in stark contrast to free, labile chemotherapeutic drugs (e.g., cisplatin). A shift in vehicle (polymers, dendrimers, nanotubes) has also seen a shift in mode of action from the conventional receptor-mediated endocytotic mechanism. pH, thermal and enzyme sensitive release and, photothermal excitation of nanoparticles are but a few novel therapeutic mechanisms. With particular focus on the latter, the chemical and physical properties of nanoparticles themselves can be exploited in cancer therapeutics [21]. Notable examples of these include micelles that form from self-assembled cyclodextrin dimers containing the Arg-Gly-Asp (RGD) sequence for target specificity, and doxorubicin as a therapeutic drug load within the inner core, which could be exploited for release by PEG removal only when exposed to a pH and temperature sensitive tumor environment [64]. Enzyme sensitive targeting has also been developed. A study by Medina *et al.* took advantage of the fact that gelatinases are highly expressed in several cancers, an indicator of poor prognosis, and modifying one of the peptide inhibitors identified by random phage display (CTT2) resulted in the formation of PEG-PE-CTT2 peptide-bound micelles, which than could be attached to PEGylated liposomes loaded with doxorubicin, creating a targeted drug delivery vehicle [65,66]. Also included is photothermal therapy, which essentially makes use of nanoshells, which have high absorption in the near infrared region. Systemic administration of the nanoparticles, as demonstrated by O’Neal *et al.*, coupled with high frequency laser excitation led to extensive thermal ablation of tumors in a mouse model [67]. Rather like precision guided ‘smart bombs’, the nanoshells, usually coated with gold, generate significant heat upon laser activation and in the process destroy the surrounding tumor area. For this reason and others, including a high functionality in biological circulation, targeted gold nanoparticles have been widely evaluated as nanoscale precision guided vehicles [68]. Intriguingly, the physical properties that aid in therapy also are being exploited for disease diagnostics. Nanoparticles can be conceivably designed so as to be detectable by microPET scanners. The simultaneous diagnosis and treatment of a disease, with easy monitoring of drug uptake and circulation would be viewed as a technological breakthrough for both patients and doctors [69].

## 5. Nanoparticle Toxicity

An ideal nanoformulation should address two main aspects of targeted delivery: high specificity to the target site and low cytotoxicity to non-diseased cells. While in principle, target specificity has been achieved by a number of nanoformulations, cytotoxicity needs to be addressed in greater depth. Both acute (short term or immediate) and chronic (long term or prolonged) toxicity of the nanomaterials has to be looked at carefully. The most common problem being faced by the research community is the *in vitro and in vivo* aggregation behavior of the various nanomaterials eventually leading to pathological and, often life threatening responses from the host. Apart from nano-aggregation-related toxicity of the nanomaterials, side effects are mainly dependent on the dosage level, surface chemistry and route of administration. Reports of toxicity of metallic nanoparticles (NP), organic NPs, and carbon nanomaterials have been published based on the above mentioned nanotoxicity criteria. However, many of these studies do not replicate the conditions in which nanoparticle DDS are employed *in vivo*, and this has led to confusion regarding the practical use of these materials in medicine [70-73]. For example, while needle-like multi-wall carbon-nanotubes longer than 20 mm show toxic effects in animals when administered by inhalation and injection, [74] shortened nanotubes (e.g., <1 mm) that are functionalized with biomolecules or PEG to provide good aqueous solubility or dispersibility show negligible cytotoxicity even at high concentrations [38,75-77]. These later studies document few short term toxicity issues in animals; however, chronic toxicity has been insufficiently studied for a range of animal models. Results suggest that shortening and adding chemical surface functionality to nanotubes can mediate toxic response [78]. These studies illustrate a strong need for comparative toxicity testing of actual nanoparticle DDS themselves, as well as for uncovering the dependence of toxicity on the actual sizes and surface functionalities of the DDS. Encouragingly, there has been considerable progress in lowering nanoparticle toxicity for imminent clinical use. As suggested above, increased dispersibility or solubility of any nanoformulation improves performance and lowers toxicity [79]. PEGylation of nanomaterials aids in reducing toxicity, helps avoid opsonization by making nanoformulations less visible to phagocytic cells, and lengthens blood circulation times [80-83]. Related risk factors regarding nanoparticle toxicity include the potential to produce CD8 and CD4 type 1 T cell responses, and it is suggested that those particles between 40 and 50 nm give rise to the maximum effect [84]. Finally, a note should be made on the need for a decision support system, for classifying nanomaterials for potential biomedical use into different risk categories, with the single goal of improving our understanding of the toxicological and potential side effects, including those parameters associated with ecotoxicity and environmental risks, and a need to identify manufacturing technology that maximizes manufacturing efficiencies and minimizes life cycle environmental risks [85,86].

## 6. Clinical Use

So far, only a handful of FDA-approved nanoparticle drugs exist. Only two—Doxil® (Centocor Ortho Biotech Products L.P) and Abraxane (Abraxis Bioscience) —address cancer therapeutics. Doxil, [87] FDA approved in 1995, was originally developed to treat HIV-related Kaposi's sarcoma, and has evolved as a second-line treatment for ovarian cancer and multiple myeloma. Doxil®, a reformulation of doxorubicin, with the drug encased in a PEGylated liposome (∼100 nM), increases its functionality and specificity while decreasing its cardiotoxicity [88]. However, the drug's tendency to concentrate in the skin induces hand-foot syndrome in over half of all patients; the redness and acutely painful peeling of the skin associated with this side-effect inhibits the complete clinical substitution of the liposomal complex for free doxorubicin [23,89]. In a similar way, Abraxane® [90] developed for treatment of breast cancer, comprises the drug paclitaxel encased in an albumin shell (∼130 nm diameter). With the secretion of SPARC (secreted protein acidic and rich in cytosine) from the tumor microenvironment, to which albumin has an affinity, the complexes become localized at the tumor site and by entering the cell through caveolae-mediated endocytosis, cancer cells undergo drug-induced destruction, resulting in a reduced tumor burden. Notable examples of nanoformulations under clinical evaluation are summarized in Table 2.

## 7. Preclinical Evaluation and Theranostics

Beyond the trials reported by Davis *et al*. in siRNA systemic delivery [57], there are several nanotechnology-enabled diagnostic and therapeutic agents currently being developed and under preclinical evaluation under the NCI Alliance for Nanotechnology in Cancer initiative (http://nano.cancer.gov/learn/now/clinical-trials.asp; [91]). These include a novel PET contrast agent family, [^18^F]-FAC, and can be used to illuminate cancerous regions of the body and help determine if patients are responding to either therapeutic drugs such as gemcitabine, cytarabine and fludarabine [95], or those in nanoformulation. Another example includes a trial led by Mirkin *et al.* at Northwestern University/ International Institute for Nanotechnology that has already received FDA approval comprises a nanosensor test for Coumadin, a drug used to prevent heart attacks, strokes, and blood clots in veins and arteries [91]. It follows that the same platform can be used to detect important cancer biomarkers, as well as measure blood levels of anticancer agents. Similarly, Ross *et al*., (MIT-Harvard Center for Cancer Nanotechnology Excellence [CCNE]), reported findings from a clinical trial determining if lymphotrophic superparamagnetic nanoparticles can be used for early detection of lymph node metastases [96]. On another front, work by Heath *et al.* (Caltech) is aimed at conducting a validation study measuring the levels of roughly 800 miRNAs from melanoma patients before and after therapy [91]. As drug companies look to develop more refined, intricate drug formulations combined with targeted delivery, current research is now moving in the direction of incorporating conventional drugs, currently freely administered, into nanocarriers. A recent representative example is the engineering of cisplatin nanoparticles that have enhanced antitumor effects and lower cytotoxicity. Paraskar *et al.* [97] developed a unique, self-assembling cisplatin nanoparticle that releases the drug in a pH-sensitive manner. The polymer to which the drug is complexed, glucosamine-functionalized polyisobutylene-maleic acid, also delivers a palliative improvement. When tested in mice, the specialized delivery noticeably reduced systemic- and nephrotoxicity, as compared with free, labile cisplatin. Dai's research group at Stanford University have carried out extensive animal studies involving carbon nanotubes where chemotherapeutic drugs were loaded onto aqueous solubilized nanotubes to efficiently target the cancer in model systems [98-103]. Our research team obtained similar results in earlier studies on cisplatin derivatized carbon nanoparticles targeted to EGF receptors [61]. Such results can potentially have global ramifications in the clinic. Whereas with free cisplatin doctors are now concerned with dosage-monitoring and buildup of platinum in the kidneys, engineered cisplatin nanoparticles herald a future where dosages can be kept minimal and toxicity greatly reduced. Notable preclinical studies on nanoformulated cancer diagnostics and therapeutics are summarized in Table 2.

Recent scientific breakthroughs including the development of biomarker initiatives have seen a paradigm shift towards personalized medicine, and with this the emergence of theranostics [19]. This evolving field of theranostics essentially refers to the combined use of therapeutic and diagnostics, with the sole purpose of optimizing efficacy and safety, and by identifying patients that are most likely to benefit from a tailored form of therapeutics. In this regard, multifunctional nanoparticle agents are now being used to integrate both therapeutics and diagnostics, with theranostics, and several are now in various stages of preclinical and clinical development [104-105]. While this nascent field has much potential in improving patient-care, there are a number of challenges that need to be overcome before it can be translated into routine clinic use [106,107]. Key among these include the development of a single platform for use in theranostics, and, elegant and sensitive methodologies to decipher the fate of nanoformulated therapeutics tailored specifically for patients, that can provide information on distribution and drug release as well as treatment efficacy after administration [108,109].

## 8. Conclusions

Thus, future challenges in nano-based DDS include (a) enhancing specificity for target cells; (b) regulating of bioavailability once these delivery systems reach the target; (c) enhancing their ability to deliver therapeutic molecules to specific sites *within* the target cells, and (d) lowering toxicity. Finally, nano-materials conjugated with target specific ligand and chemo-therapeutic drugs have the potential to selectively kill cancer cells leaving healthy non-diseased cells intact, and with PEGylation, these complexes can be rendered more hydrophilic and aqueous-dispersible, allowing for a prolonged desired effect and without toxicity.

## Figures and Tables

**Table 1.** Examples of different nanomaterial platforms available for use in drug delivery systems (DDS).

***Platform***	***Characteristics***	***References***
Liposomes	Drug encapsulation, hydrophilic interior, individual lipids can be changed to accommodate particular functionality (surface charge, *etc*).	[12,22,23]
Dendrimers	Large number of peripheral functional groups allows for the multiple drug, label, ligand functionalization.	[24-26]
Polymers	Most widely used drug delivery vehicles, some are self- assembling, can be coated with solubilizing agents, non-immunogenic and highly versatile.	[27-29]
Metallic particles	Generally used as diagnostic agents, drug delivery, thermal-ablation via laser excitation, multifunctional.	[10,19,30]
Carbon nanotubes	High functionality, limited solubility, functionalized CNT acts as an inert bioconjugate *in vivo*, drug delivery “missiles”.	[31-34]
Lipoproteins	Biocompatible protein-lipid based molecules which can carry hydrophobic drugs to tumor targets with minimal toxicity.	[35,36]

**Table 2.** Nanoformulations in preclinical and clinical evaluation.

***Preclinical***	***Features***	***References***
Kipps *et al.*	Stimulation of the immune system using a chemically engineered adenovirus nanoparticle in the treatment of leukemia	[91]
Davis *et al.*	Delivery of siRNA using cyclodextrin-based nanoparticles in cancer treatment	[91]
Davis *et al.*	Administration of camptothecin bound to cyclodextrin-based polymer for treatment of solid tumors	[91]
Mirkin *et al.*	Detection of drug levels in body using nanosensors, applicable also to cancer biomarkers	[91]
Heath *et al.*	Measurement of miRNA levels in melanoma patients pre- and post-treatment using the Integrated Blood Barcode (IBBC) chip	[91]
Langer and Farokhzad	Multifunctional drug delivery using a polymer matrix, therapeutic payload(s), surface moieties, and targeting ligands	[91]
***Clinical***	***Features***	***References***
Shimada *et al.*	INGN-201 (Vivante GMP Solutions) a liposomal nanoformulation for lung cancer treatment administered intravenously is in Phase I.	[92,93]
Schwartz *et al.*	AuraShell (Nanospectra Biosciences) a gold-coated silica NP based drug formulation for treatment of solid tumors, now under Phase I evaluation.	[94]
